# Temporal Machine Learning Analysis of Prior Mammograms for Breast Cancer Risk Prediction

**DOI:** 10.3390/cancers15072141

**Published:** 2023-04-04

**Authors:** Hui Li, Kayla Robinson, Li Lan, Natalie Baughan, Chun-Wai Chan, Matthew Embury, Gary J. Whitman, Randa El-Zein, Isabelle Bedrosian, Maryellen L. Giger

**Affiliations:** 1 Department of Radiology, The University of Chicago, Chicago, IL 60637, USA; huili@uchicago.edu (H.L.);; 2 Department of Breast Surgical Oncology, The University of Texas MD Anderson Cancer Center, Houston, TX 77030, USA; 3 Department of Breast Imaging, The University of Texas MD Anderson Cancer Center, Houston, TX 77030, USA; 4 Department of Radiology, Houston Methodist Research Institute, Houston, TX 77030, USA

**Keywords:** breast cancer risk, radiomics, long short-term memory networks, artificial intelligence, field effect

## Abstract

**Simple Summary:**

Machine learning approaches, using both radiomic and deep-learning-based features, were performed for an analysis of the breast parenchyma to identify women at risk of future breast cancer. Results from this study demonstrate that the antecedent mammographic images can potentially discriminate between women with a future-biopsy-proven cancer versus those with a future-biopsy-proven benign lesion.

**Abstract:**

The identification of women at risk for sporadic breast cancer remains a clinical challenge. We hypothesize that the temporal analysis of annual screening mammograms, using a long short-term memory (LSTM) network, could accurately identify women at risk of future breast cancer. Women with an imaging abnormality, which had been biopsy-confirmed to be cancer or benign, who also had antecedent imaging available were included in this case–control study. Sequences of antecedent mammograms were retrospectively collected under HIPAA-approved guidelines. Radiomic and deep-learning-based features were extracted on regions of interest placed posterior to the nipple in antecedent images. These features were input to LSTM recurrent networks to classify whether the future lesion would be malignant or benign. Classification performance was assessed using all available antecedent time-points and using a single antecedent time-point in the task of lesion classification. Classifiers incorporating multiple time-points with LSTM, based either on deep-learning-extracted features or on radiomic features, tended to perform statistically better than chance, whereas those using only a single time-point failed to show improved performance compared to chance, as judged by area under the receiver operating characteristic curves (AUC: 0.63 ± 0.05, 0.65 ± 0.05, 0.52 ± 0.06 and 0.54 ± 0.06, respectively). Lastly, similar classification performance was observed when using features extracted from the affected versus the contralateral breast in predicting future unilateral malignancy (AUC: 0.63 ± 0.05 vs. 0.59 ± 0.06 for deep-learning-extracted features; 0.65 ± 0.05 vs. 0.62 ± 0.06 for radiomic features). The results of this study suggest that the incorporation of temporal information into radiomic analyses may improve the overall classification performance through LSTM, as demonstrated by the improved discrimination of future lesions as malignant or benign. Further, our data suggest that a potential field effect, changes in the breast extending beyond the lesion itself, is present in both the affected and contralateral breasts in antecedent imaging, and, thus, the evaluation of either breast might inform on the future risk of breast cancer.

## 1. Introduction

While agencies such as the American College of Radiology, American College of Physicians, and American Cancer Society have different recommendations for breast screening frequency guidelines, they all suggest mammographic screening with some frequency over some portion of a woman’s lifetime [1,2,3]. Women who follow these guidelines produce, over the years, temporal sequences of mammographic images. When interpreting screening exams, radiologists often compare current mammograms with prior mammograms to qualitatively assess the interval change in breast tissue. Such a practice is conducted because the interval change may indicate the development of a new cancer [4].

It has been demonstrated that comparing current and prior mammograms improves specificity in breast cancer screening. A study that compared performance on over one million images found that the use of comparison mammograms at screening resulted in lower recall rates (6.9% with comparison mammograms vs. 14.9% without comparison mammograms) and higher specificity (93.5% with comparison mammograms vs. 85.7% without comparison mammograms) [4]. This suggests that in ambiguous cases, where it is not obvious whether an abnormality poses a threat, the changes in mammograms over time provide the radiologist with discriminatory information that helps inform the decision of whether or not to send a patient for follow-up. For example, if a suspicious region is judged to be visible and unchanged from prior mammograms, then the risk of malignancy may be lower as evaluated by the radiologist. The utility of prior images in radiologist review suggests the incorporation of prior images may also be informative in artificial-intelligence-based cancer prediction systems aimed at assisting the radiologist in detecting cancer risk.

A number of studies have shown the utility of incorporating prior imaging exams in clinical classification tasks. A study by Santeramo et al. [5] implemented a time-modulated long short-term memory (LSTM) network to detect abnormalities in a database of 745,480 chest X-rays, with the intent to classify abnormalities as either cardiomegaly, consolidation, pleural effusion, or hiatus hernia. The study compared the performance of a convolutional neural network (CNN Inception v3) trained on single images as a baseline to an LSTM network using the single images plus prior longitudinal observations. Using the F-measure as a figure of merit, the study observed, on average over the four abnormality types, that the LSTM resulted in a 7% increase in F-measure and a 9% increase in PPV over the baseline, single-image CNN. A study by Shao et al. [6] investigated the use of temporal radiomics to interrogate normal appearing white matter (NAWM) in order to predict the development of white matter hyperintensities (WMH) which are associated with cognitive decline among elderly patients. This study constructed radiomic signatures on regions of interest among a cross sectional cohort of cases with noted progression of WMH and aged-matched controls without progression to WMH, each of which had undergone two or more MRI exams on the same scanner with a time period of at least one year between scans. The study reported an area under the curve (AUC) of 0.954 (95% confidence interval: 0.876–0.989) for distinguishing between areas of NAWM that developed into WMH from those that did not develop into WMH. In addition, in predicting the malignancy of breast lesions on dynamic contrast-enhanced magnetic resonance images (MRI), LSTM has been used to incorporate the multiple acquisition time-points within the dynamic imaging protocol [7]. Specifically, Antropova et al. demonstrated higher classification performance on lesion characterization with MRI using LSTM than using a fine-tuned feed-forward network at a single time-point [7]. These studies provide evidence that a computer analysis of temporal images may improve the accuracy of predicting future disease.

Given the relevance of serial imaging in the diagnostic interpretation of mammographic findings and the emerging findings on the importance of incorporating temporal data for the classification of a future disease state, we sought to test the hypothesis that a computer analysis of multiple sequential antecedent mammograms could predict the future risk of benign versus malignant breast lesions. Our study investigates both conventional human-engineered radiomic features and deep learning methods for the task of classifying future lesions.

In order to incorporate information collected over a time series of full field digital mammograms (FFDMs), we chose to use an LSTM network in this study, as it is capable of learning long-term dependencies for data organized as a series [8]. As a recurrent neural network (RNN), LSTM networks are able to retain information about previous time-points in a series and use this information to inform decisions on the present time-points of that same series [9,10]. LSTM networks can take in feature vectors from various sources, and so this study explored the performance of an LSTM trained on features extracted from a CNN and the performance of an LSTM trained on conventional human-engineered features extracted from the same images. Additionally, we measured the performance obtained by extracting features from a single time-point and merging features using a support vector machine (SVM) classifier. In this way, an assessment was performed between deep features and conventional human-engineered features as well as between time series data and single-time-point data for classification.

## 2. Materials and Methods

### 2.1. Image Acquisition and Database Description

Mammograms were retrospectively collected from MD Anderson Cancer Center and the University of Chicago Medical Center of women who had undergone screening exams for two or more years prior to the detection of a mammographic abnormality. Subjects identified at MD Anderson were part of a cohort of women recruited prospectively evaluating blood and tissue biomarkers of breast cancer risk; the subset of subjects with prior mammograms was included in this analysis. Subjects at the University of Chicago were identified retrospectively from an imaging database of women undergoing both screening and diagnostic mammograms. Images were acquired between 2006 and 2019 and were collected for this analysis in compliance with the Health Insurance Portability and Accountability Act (HIPAA) and under institutional-review-board-approved protocols at each institution.

For each patient exam, the CC images of the left and right breast were used in analysis. Each patient included in this study had ultimately undergone core biopsy of an imaging abnormality with histopathologically confirmed findings of a malignant or benign lesion. However, it is important to note that all the images analyzed in this study were acquired prior to the detection of each mammographic abnormality (i.e., were antecedent images). The laterality of each mammographic abnormality was noted, and the affected and contralateral breasts were treated separately in the analyses.

**Figure 1:**
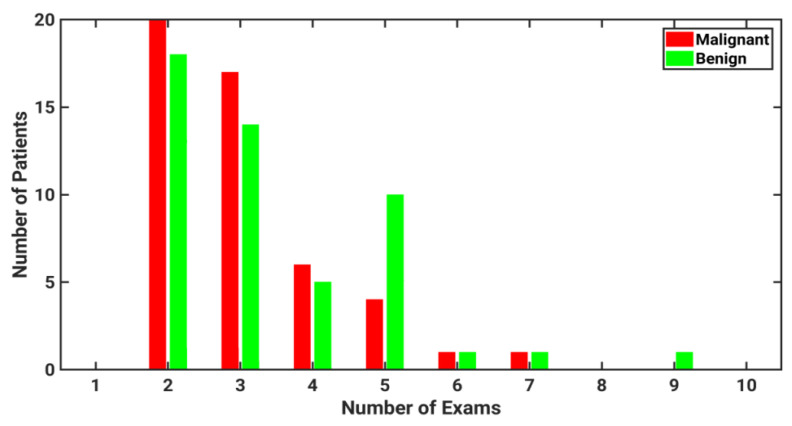
Histogram of the number of mammographic exams included in the study for patients with either malignant or benign lesions. All images included were acquired prior to the screening exam that ultimately led to diagnosis.

**Figure 2:**
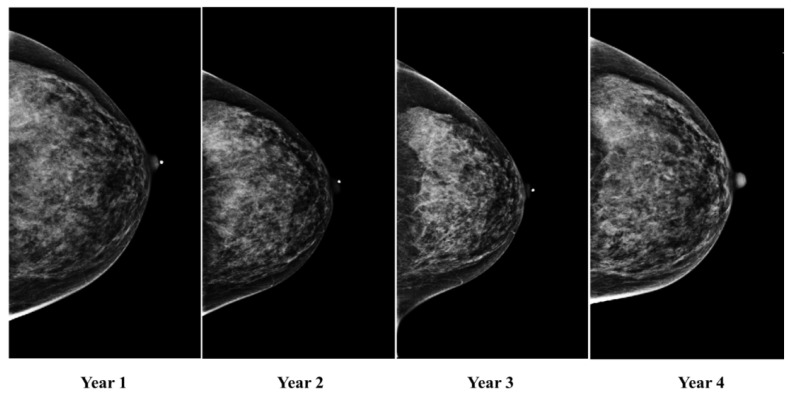
Temporal mammograms for one patient, collected annually over a span of four years.

The number of prior mammographic exams per participant ranged from 2 to 9 (Figure 1). Note that the period of time between subsequent screening exams was not always constant for each patient. The average time between exams was 1.27 years. The temporal mammograms for one patient, collected annually over a span of four years, are shown in Figure 2.

A total of 318 mammographic exams from 99 patients were included in the study. Of these, 49 patients were eventually diagnosed with a malignant finding and 50 were diagnosed with a benign finding. The mean age was 57.6 years (standard deviation 9.4 years) for the 49 cancer patients and 54.6 years (standard deviation 8.8 years) for the 50 cancer-free controls. All images were acquired on Hologic systems with pixel sizes of 70 μm × 70 μm and were processed according to the clinical standard at the patient’s screening institution.

### 2.2. Radiomic Feature Extraction

Computer-extracted radiomic features were automatically calculated on square ROIs of size 512 × 512 pixels, which had been manually placed in the central breast region posterior to the nipple. From each region, 50 features were automatically extracted and are summarized in Table 1. Additional details of these mathematical descriptors from feature extraction have been described elsewhere [11,12,13,14,15,16]. Features were selected to describe the intensity and spatial pattern of the texture in each image region.

### 2.3. Deep Feature Extraction

Deep-learning-based feature extraction was performed on the same ROIs used for radiomic feature calculation. Features were extracted using the pre-trained VGG-19 neural network [17]. A total of 1472 features were extracted from each image using the neural network. Features were extracted from each max pooling layer of the network, and an additional average pooling layer was added to reduce the dimensionality of the features. This approach of transfer learning has been studied and implemented elsewhere [18,19,20].

### 2.4. Long Short-Term Memory Network

Recurrent neural networks (RNNs) are designed for making classifications and predictions based on a time series of data [10]. RNNs are composed of a series of identical feed-forward neural networks. In this series of networks, each individual network is used to analyze a single time-point and is known as an RNN cell. Each RNN cell produces a recurrent output that is passed on to the next time step. Likewise, each RNN cell accepts a prior state as input. In this way, information from prior time-points informs the output of future time-points.

Mathematically, an RNN cell can be represented by Equation (1), where *s_t_* is the current state, *s_t−_*_1_ is the prior state, *x_t_* is the current input, and *f* is the recurrent function. Thus, a basic single-layer RNN can be written as in Equation (2), where *ϕ* is the activation function, and *W*, *U*, and *b* are the weights and biases of the network.

(1) $$\left(\begin{array}{c} s_{t}\\ o_{t} \end{array}\right)=f\left(\begin{array}{c} s_{t-1}\\ x_{t} \end{array}\right)$$

(2) $$s_{t}=\phi \left(Ws_{t-1}+Ux_{t}+b\right)$$

**Figure 3:**
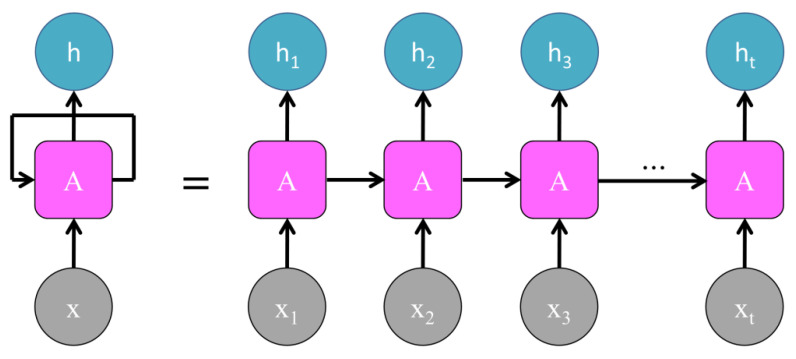
General architecture of an RNN cell component, where A represents the neural network, *x_t_* represents the input, and *h_t_* represents the output value [8].

The general recurrent structure of an RNN is illustrated in Figure 3, where it is shown that information from the RNN cell for one time-point in the series is passed along to the cell for the next input from the series.

In order to avoid the potential pitfalls of information morphing and of the vanishing gradient problem, LSTM cells are designed to contain three gates that are not typically present in conventional RNNs: the input gate, output gate, and forget gate. These three gates monitor the extent to which information is read in from an adjacent time-point, how much of this information to write out, and to what extent the information is remembered and passed on to the next time-point. The input gate (*i_t_*), output gate (*ο_t_*), and forget gate (*f_t_*) are defined as:

(3) $$i_{t}=\sigma \left(W_{i}s_{t-1}+U_{i}x_{t}+b_{i}\right)$$

(4) $$o_{t}=\sigma \left(W_{o}s_{t-1}+U_{o}x_{t}+b_{o}\right)$$

(5) $$f_{t}=\sigma \left(W_{f}s_{t-1}+U_{f}x_{t}+b_{f}\right)$$

where *s_t_*_−1_ is the prior state, *x_t_* is the current input, *σ* is the sigmoid function, and *W*, *U,* and *b* are the weights and biases of the network.

### 2.5. Classification and Evaluation

In order to evaluate the value of temporal information relative to single time-point analysis, classifications were performed using both SVM (single time-point) and LSTM (multiple time-points) in the task of predicting the histologic diagnosis of future lesions. The same feature set was used for training the LSTM and SVM networks. In this experiment, we decided to use SVM for comparisons as opposed to a feed-forward network in order to reduce the likelihood of overfitting. To characterize repeatability, 5-fold cross validation was used for each classifier, with folds kept consistent over each classifier along with the same proportions of malignant and benign cases in each fold. This method ensured that training and testing splits were kept consistent for pairwise comparisons between classifiers. Each classifier was trained separately on the antecedent images of the affected and contralateral breasts in the task of classifying the histologic diagnosis (cancer versus benign) of a future lesion. Note that images of any given case were kept together in either the training or testing fold.

ROI placement and radiomic feature extraction were performed on a dedicated workstation developed in our lab [12,13,14,15]. CNN feature extraction and network training were performed in Keras (Version 2.1.2) using a TensorFlow (Version 1.10.0) backend framework [21,22].

### 2.6. Temporal Sequence Classification with LSTM Network

**Figure 4:**
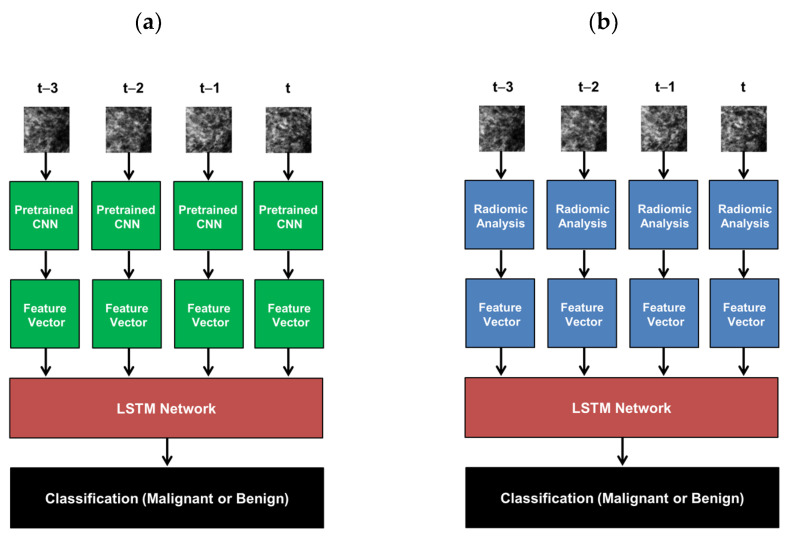
Summary of the workflow involved in using LSTM networks to classify temporal sequences of mammograms in this study, where t is the time of the most recent mammogram included in analysis. (**a**) Workflow for CNN-extracted features, and (**b**) workflow for radiomic features. Classifications were performed to predict the probabilities of future malignant lesions based only on antecedent images.

In order to evaluate classification performance with the inclusion of multiple mammographic time-points, features extracted from each image were used as inputs to the LSTM network. To consider the value of the human-engineered radiomic features compared with the CNN features, separate networks were trained using each of these two as input features, as illustrated in Figure 4. Each classifier described was trained in the task of classifying future lesions as malignant or benign using only the prior antecedent images.

The LSTM network in this study was trained using a stochastic gradient descent (SGD) optimizer [23]. In SGD, optimal weights are determined by choosing a random sample of training vectors and using these to compute an estimate of the gradient at each step of the training procedure. Given a random batch of training objects, the update by SGD is given by Equation (6), where *θ* is the parameter to update, *α* is the learning rate, *J* is the objective function, and (*x*^(*i*)^,*y*^(*i*)^) are the training feature vectors.

(6) $$\theta =\theta -\alpha \nabla_{\theta }J\left(\theta ;x^{\left(i\right)},y^{\left(i\right)}\right)$$

Hyperparameters were selected by performing a limited sweep of learning rate and hidden dimension parameters. After sweeping over hidden dimensions of 512, 1024, and 2048, and sweeping over learning rates of 10^−3^, 10^−4^, and 10^−5^, the combination of parameters of hidden dimension of 512 and learning rate of 10^−4^ was selected for the task of classifying future malignant lesions using antecedent images. Since each patient had a different number of images across the dataset, the feature sequences were padded with zeros to the length of the longest sequence, as typically conducted with LSTM. The padded part of the sequences was not taken into account when calculating the binary cross-entropy loss of the model [7]. For each LSTM network, 100 epochs were used in training.

### 2.7. Single Time-Point Classification with Support Vector Machine

To understand further the effect of multiple time-points, classification was also performed using the image collected only one year prior to diagnosis in the task of classifying the likelihood of malignancy of the future lesion. As only one single time-point is used for classification, a 5-fold cross validation using a support vector machine (SVM) as a classifier was performed [24]. To reduce dimensionality, principal component analysis (PCA) was performed to reduce the feature space to 25 principal components prior to training the SVM [25]. Training and classification were performed using the human-engineered radiomic features as an input, as well as using features extracted by the pretrained CNN as an input.

### 2.8. Statistical Evaluation

From receiver operating characteristic (ROC) analysis, the area under the curve (AUC) was used as a figure of merit in the task of predicting malignancy using antecedent images, and the statistical difference between the AUC values for different models was computed using ROCKIT software [26,27]. Corrections were made for multiple comparisons following the Holm–Bonferroni correction [28].

## 3. Results

The performance of each classification model for distinguishing a future benign state from a future malignant state is summarized in Table 2. In general, classifiers incorporating multiple time-points with LSTM, based either on deep-learning-extracted features or on radiomic features, tended to perform statistically better than chance (AUC = 0.5), whereas those using only a single time-point failed to show improved performance compared to chance, as judged by the area under the receiver operating characteristic curves (AUC: 0.63 ± 0.05, 0.65 ± 0.05, 0.52 ± 0.06 and 0.54 ± 0.06, respectively) for each of the affected breast and similarly for each of the contralateral breast (AUC: 0.59 ± 0.06, 0.62 ± 0.06, 0.52 ± 0.06 and 0.55 ± 0.06, respectively).

Note that we failed to show a significant difference in the AUC between the LSTM network trained using CNN-extracted features and that trained using radiomic features in the task of classifying future lesions as malignant or benign. This trend held for both classifications using the affected breast (AUCs of 0.63 vs. 0.65, *p* = 0.6511, 95% CI of ΔAUC [−0.1631, 0.1019]) and using the contralateral breast (AUCs of 0.59 vs. 0.62, *p* = 0.8083, 95% CI of ΔAUC [−0.1743, 0.1359]).

In clinical practice, it is unknown whether a future lesion will develop in the right or left breast. Therefore, it is more clinically relevant to examine a merged classifier, which takes into account the classifier output on each the left and right breast in the task of predicting whether the future lesion will be malignant or benign. We failed to demonstrate significant difference between classifiers trained using CNN features extracted from affected and contralateral breasts (AUCs of 0.63 vs. 0.59, *p* = 0.7278, 95% CI of ΔAUC [−0.0898, 0.1286]) and radiomic features extracted from affected and contralateral breasts (AUCs of 0.65 vs. 0.62, *p* = 0.6273, 95% CI of ΔAUC [−0.0211, 0.0350]).

**Figure 5:**
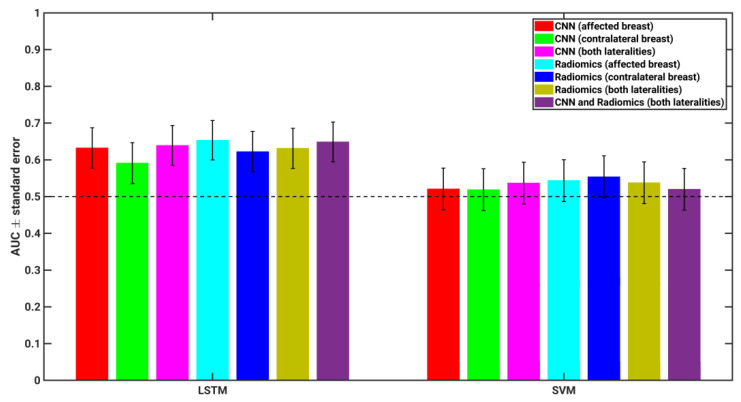
AUC values for each classifier compared, including merged classifiers. Each merged classifier was constructed by taking the average classifier output from two different classifiers for each individual case, and then performing ROC analysis on the averaged output values in the task of characterizing future lesions as malignant or benign. Error bars show one standard error. Dashed line is classification performance with guessing (AUC = 0.5).

Furthermore, it is also of interest to explore the classification performance in the task of characterizing future lesion malignancy when both the human-engineered and deep learning methods were combined over both breasts, as presented in Table 2 and Figure 5. Statistical comparisons were not performed on the merged classifier output in order to maintain statistical power by limiting the quantity of pairwise comparisons performed.

## 4. Discussion

The results from this study demonstrated that LSTM classifiers using multiple time-points, based either on deep-learning-extracted features or on radiomic features, tended to perform statistically better than chance, whereas those using only a single time-point failed to show improved performance compared to chance, as judged by the area under the receiver operating characteristic curves (AUC: 0.63 ± 0.05, 0.65 ± 0.05, 0.52 ± 0.06 and 0.54 ± 0.06, respectively). The classification performance in the task of predicting future lesion malignancy was not observed to be statistically significantly different when an LSTM network was trained using either CNN features or using radiomic features. This suggests that, while these feature sets are different in their origin and how they are extracted, they achieve similar results. Thus, either feature set may be appropriate for classifications with temporal LSTM networks.

Similar classification performance was observed between the performance using features extracted from the affected and contralateral breast in predicting the malignancy of future unilateral lesions. Because only antecedent images were used in this analysis, no mammographic abnormalities were present. Thus, while it is possible that the affected breast had a precancerous texture change leading up to lesion detection, these results suggest that a change also occurred in the contralateral breast that may indicate future malignancy. Thus, this observation suggests that a field effect, changes in the breast extending beyond the lesion itself, is present in both the affected and contralateral breasts in antecedent imaging and, thus, the evaluation of either breast is informative for cancer risk assessment.

This investigation into the use of temporal sequences of data for malignancy prediction has several limitations. First, this study used a dataset of limited size compared with other implementations of LSTM networks. The curation of large datasets is more challenging and expensive in the medical domain compared with natural images, thus resulting in our small number of cases included. Additionally, the data used in this study were collected at two separate institutions, with slightly different cancer prevalence rates in the corresponding datasets. While all images were acquired on Hologic units, differences in image acquisition procedures may have varied between the two medical centers, resulting in some differences in image characteristics.

Additionally, the intervals at which women underwent screening were not consistent. While national agencies suggest screening at regular intervals of time, patient compliance was not consistent in the data. Furthermore, women may have undergone screening at an institution outside of the two involved in this study, and, therefore, this additional image was omitted from this investigation. Collecting images from consistent time intervals may affect, and potentially improve, the performance observed in this study.

The nature of screening exams involves repeat imaging on separate exam dates, thus inherently involving the repositioning of the patient in the imager. As a result of this, images are not spatially registered to one another. While this may be solved through deformable registration methods, it is likely that such image processing would alter the radiomic features extracted, potentially reducing the efficacy of such features. The approach taken in this study was to manually align ROIs on undeformed images; however, this method only results in approximate spatial registration across exam dates. While previous studies have shown that radiomic features tend to be only minimally impacted by small changes in spatial placement of an ROI, there may still be some effect present [29].

Finally, note that this study compared a new method, using LSTM networks to incorporate temporal information, with a conventional supervised learning approach (SVM) that does not involve deep learning. The transfer learning approach of using SVM to merge CNN-extracted image features has also shown promise in other FFDM studies [18,30].

This paper presents an image-based breast cancer prediction method that captures temporal information about parenchymal texture on FFDM over time. These temporal sequences are used to classify future lesions as either malignant or benign.

Compared with the previous methods, this work allowed for the incorporation of imaging information from multiple antecedent images, as opposed to just a single image. Thus, this method evaluated not only the appearance of the parenchyma, but also changes in the parenchyma over time. This work explored temporal network performance when using features extracted either by conventional radiomics methods and from the pre-trained VGG-19 network.

Based on the analyses performed in this study, LSTM networks based either on deep-learning-extracted features or on radiomic features from either affected breast or contralateral breast tended to perform statistically better than chance, whereas those using only a single time-point failed to show improved performance compared to chance.

The main motivation for the selection of LSTM networks for use in characterizing temporal image sequences is their ability to prevent vanishing or exploding gradients during error backpropagation. Additionally, LSTM networks are well suited to handle sequences of varying length, as women have varying numbers of screening mammograms throughout their lifetimes.

The method used in this study was motivated by the fact that human experts compare current screening mammograms with previous screening mammograms to assist in the detection of abnormality. This suggests that prior images may provide additional information to the current image [4]. Thus, changes in texture over time may be indicative of an elevated probability of developing a malignant breast lesion.

The deep learning methods employed here captured temporal data patterns that are not typically examined in conventional radiomics approaches. This work has shown that the temporal data patterns of either breast capture clinically useful information in evaluating the classification of future lesions based on screening mammography.

## 5. Conclusions

A long short-term memory (LSTM) network for the analysis of breast parenchyma, using both radiomic and deep-learning-based features, was performed to identify women predisposed to developing breast cancer. The findings from this study demonstrated that the incorporation of temporal information into radiomic analyses may improve overall classification performance through LSTM, as demonstrated by the improved discrimination of future lesions as malignant or benign. Further, our data suggest that a potential field effect, changes in the breast extending beyond the lesion itself, is present in both the affected and contralateral breasts in antecedent imaging, and, thus, the evaluation of either breast might inform on the future risk of breast cancer.

## Figures and Tables

**Table 1:** Summary of features included for analysis in the radiomics feature set.

Feature Category	Number of Features
Box counting fractal dimension	6
Edge gradient	4
Histogram	10
Fourier	2
Neighborhood Gray-Tone Difference Matrix	5
Minkowski fractal dimension	1
Powerlaw beta	8
GLCM	14
Total	50

**Table 2:** Performance of each classification model for distinguishing future benign state from future malignant state.

Feature Type	LSTM Classifier AUC (*p*-Value) * [95% CI of AUC]	SVM Classifier AUC (*p*-Value) * [95% CI of AUC]
CNN (affected breast)	AUC = 0.63 (*p* = 0.0231) [0.5010, 0.7175]	AUC = 0.52 (*p* = 0.7103) [0.3962, 0.6193]
CNN (contralateral breast)	AUC = 0.59 (*p* = 0.1024) [0.4791, 0.6982]	AUC = 0.52 (*p* = 0.7389) [0.4083, 0.6320]
CNN (both lateralities)	AUC = 0.64 (*p* = 0.0104) [0.5184, 0.7336]	AUC = 0.54 (*p* = 0.5140) [0.4138, 0.6372]
Radiomics (affected breast)	AUC = 0.65 (*p* = 0.0042) [0.5346, 0.7456]	AUC = 0.54 (*p* = 0.4425) [0.4510, 0.6723]
Radiomics (contralateral breast)	AUC = 0.62 (*p* = 0.0259) [0.4998, 0.7161]	AUC = 0.55 (*p* = 0.3434) [0.4439, 0.6672]
Radiomics (both lateralities)	AUC = 0.63 (*p* = 0.0159) [0.5122, 0.7263]	AUC = 0.54 (*p* = 0.5035) [0.4216, 0.6454]
CNN + Radiomics (both lateralities)	AUC = 0.65 (*p* = 0.0059) [0.5109, 0.7282]	AUC = 0.52 (*p* = 0.7226) [0.4190, 0.6422]

* *p*-value is estimated using z-score test by comparing classifier performance with chance (AUC = 0.5). CI: confidence interval.

## Data Availability

Radiomic data are available upon reasonable request to the authors.
